# Supplementary material for: Causal Relationship between Aging and Anorexia Nervosa: A White-Matter-Microstructure-Mediated Mendelian Randomization Analysis
Source: Biomedicines. 2024 Aug 16;12(8):1874. doi: 10.3390/biomedicines12081874 (PMC11351342; doi:10.3390/biomedicines12081874)
Supplement: Supplementary file 1 [file biomedicines-12-01874-s001.zip › Supplementary Figure/Figure S4/Singe SNP plot of MR analysis for TL to ubm-b-2114.pdf]

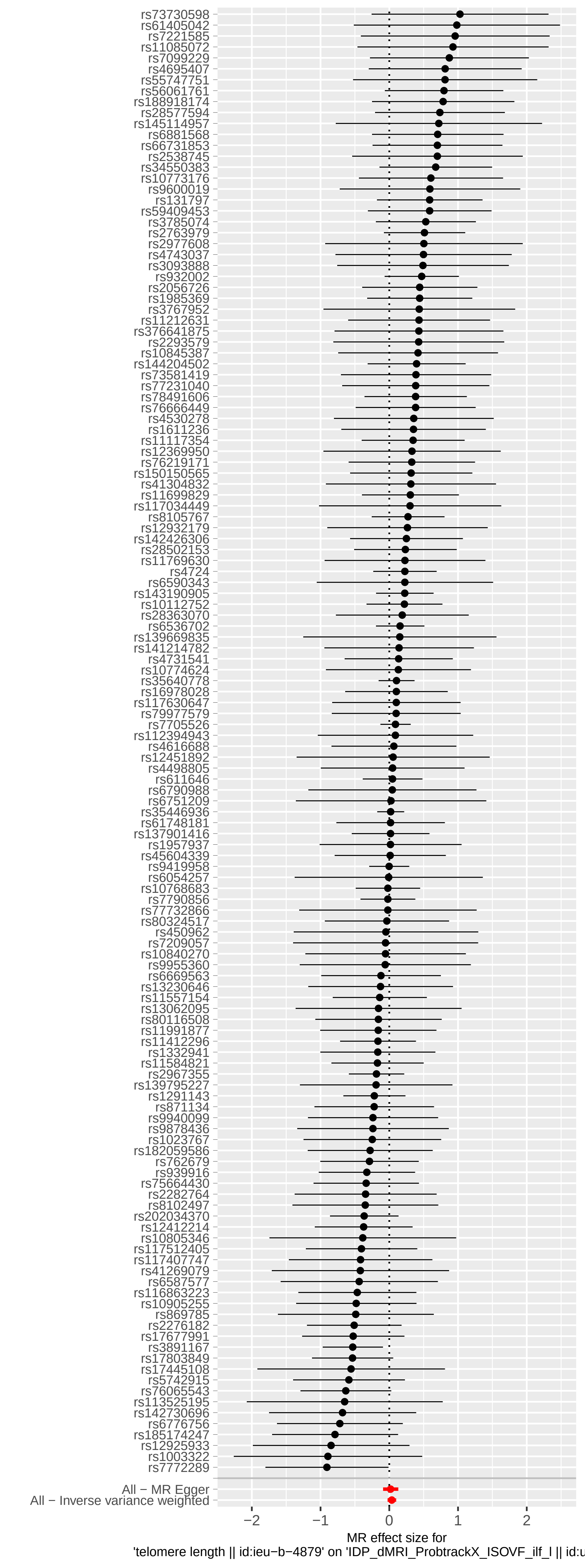
rs73730598
rs61405042
rs7221585
rs11085072
rs7099229
rs4695407
rs55747751
rs56061761
rs188918174
rs28577594
rs145114957
rs6881568
rs66731853
rs2538745
rs34550383
rs10773176
rs9600019
rs131797
rs59409453
rs3785074
rs2763979
rs2977608
rs4743037
rs3093888
rs932002
rs2056726
rs1985369
rs3767952
rs11212631
rs376641875
rs2293579
rs10845387
rs144204502
rs73581419
rs77231040
rs78491606
rs76666449
rs4530278
rs1611236
rs11117354
rs12369950
rs76219171
rs150150565
rs41304832
rs11699829
rs117034449
rs8105767
rs12932179
rs142426306
rs28502153
rs11769630
rs4724
rs6590343
rs143190905
rs10112752
rs28363070
rs6536702
rs139669835
rs141214782
rs4731541
rs10774624
rs35640778
rs16978028
rs117630647
rs79977579
rs7705526
rs112394943
rs4616688
rs12451892
rs4498805
rs611646
rs6790988
rs6751209
rs35446936
rs61748181
rs137901416
rs1957937
rs45604339
rs9419958
rs6054257
rs10768683
rs7790856
rs77732866
rs80324517
rs450962
rs7209057
rs10840270
rs9955360
rs6669563
rs13230646
rs11557154
rs13062095
rs80116508
rs11991877
rs11412296
rs1332941
rs11584821
rs2967355
rs139795227
rs1291143
rs871134
rs9940099
rs9878436
rs1023767
rs182059586
rs762679
rs939916
rs75664430
rs2282764
rs8102497
rs202034370
rs12412214
rs10805346
rs117512405
rs117407747
rs41269079
rs6587577
rs116863223
rs10905255
rs869785
rs2276182
rs17677991
rs3891167
rs17803849
rs17445108
rs5742915
rs76065543
rs113525195
rs142730696
rs6776756
rs185174247
rs12925933
rs1003322
rs7772289
All − MR Egger
All − Inverse variance weighted
−2
−1
0
1
2
MR effect size for
'telomere length || id:ieu−b−4879' on 'IDP_dMRI_ProbtrackX_ISOVF_ilf_l || id:u
